# Detection of subtelomere imbalance using MLPA: validation, development of an analysis protocol, and application in a diagnostic centre

**DOI:** 10.1186/1471-2350-8-9

**Published:** 2007-03-05

**Authors:** Joo Wook Ahn, Caroline Mackie Ogilvie, Alysia Welch, Helen Thomas, Rajiv Madula, Alison Hills, Celia Donaghue, Kathy Mann

**Affiliations:** 1 Cytogenetics Department, Guy's and St Thomas' NHS Foundation Trust, London, UK; 2 Department of Medical and Molecular Genetics, King's College London School of Medicine, Guy's Hospital, London, UK

## Abstract

**Background:**

Commercial MLPA kits (MRC-Holland) are available for detecting imbalance at the subtelomere regions of chromosomes; each kit consists of one probe for each subtelomere.

**Methods:**

For validation of the kits, 208 patients were tested, of which 128 were known to be abnormal, corresponding to 8528 genomic regions overall. Validation samples included those with trisomy 13, 18 and 21, microscopically visible terminal deletions and duplications, sex chromosome abnormalities and submicroscopic abnormalities identified by multiprobe FISH. A robust and sensitive analysis system was developed to allow accurate interpretation of single probe results, which is essential as breakpoints may occur between MLPA probes.

**Results:**

The validation results showed that MLPA is a highly efficient technique for medium-throughput screening for subtelomere imbalance, with 95% confidence intervals for positive and negative predictive accuracies of  0.951-0.996 and 0.9996-1 respectively. A diagnostic testing strategy was established for subtelomere MLPA and any subsequent follow-up tests that may be required. The efficacy of this approach was demonstrated during 15 months of diagnostic testing when 455 patients were tested and 27 (5.9%) abnormal cases were detected.

**Conclusion:**

The development of a robust, medium-throughput analysis system for the interpretation of results from subtelomere assays will be of benefit to other Centres wishing to implement such an MLPA-based service.

## Background

Genomic imbalance at the subtelomeres of chromosomes has been found in individuals with idiopathic mental retardation, dysmorphic features and/or congenital abnormalities. The reported incidence is dependent on referral group: Joyce *et al *[1] found no imbalance in 200 patients with idiopathic mental retardation but without dysmorphism; whilst Rio *et al *[2] report that 10.7% of patients with severe idiopathic mental retardation were found to have subtelomere imbalance. Data collated from 20 studies found that of 2,500 patients with mental retardation, 4.8% were found to have deletion or duplication of subtelomeric sequences [3] and the investigation of an impressive cohort (11,688 cases) by Ravnan *et al *[4], found 2.5% had clinically significant subtelomere imbalance, with most referred for developmental delay with or without dysmorphic features. This prevalence indicates that testing would be justified in appropriate referral groups. However, until recently the diagnostic potential of subtelomere testing has been limited by the technology available, with the majority of diagnostic centres employing a laborious and expensive multiprobe FISH approach [5].

The development and availability of multiplex ligation-dependent probe amplification (MLPA) for the accurate assessment of copy number at multiple loci has provided a new approach for subtelomere testing. This elegant technique can assess 48 loci in a single reaction and lends itself to cost-effective medium-throughput testing. An impressive number of MLPA studies has been published since the technique was first described in 2002 [6]. The majority of these studies have used commercial MLPA assays (MRC-Holland) to detect deletions and duplications of exons causing single gene disorders [7-9]. However, most cases of submicroscopic subtelomere imbalance involve duplication or deletion of a single subtelomere and therefore in current, commercial assays, a single MLPA probe. This is in contrast to MLPA testing for single gene disorders, where contiguous exon aberrations generally involve multiple MLPA probes. Furthermore, exon testing-MLPA assays incorporate designated control probes from outside of the tested region; commercial subtelomere assays do not contain any such designated control probes and any probe may show a change in copy number. Thus the strategy used to analyse MLPA data for the detection of subtelomere imbalance needs to be shown to produce robust single probe results in the absence of control probes. Several analytical models have been proposed [10-12] and five studies [10-14] using these analysis models are summarised in Table 1. The abnormality detection rate ranged from 1.8% to 5.9% and the detection rate for inherited abnormalities or polymorphisms ranged from 0% to 6.2% (it should be noted that some of the probesets used in these studies are now known to contain probes that detect polymorphic loci). Further studies have demonstrated higher detection rates, especially if more stringent clinical criteria are used to select the patient cohort [10,15,16].

**Table 1:** A summary of published results using MLPA to identify subtelomere imbalance.

**Report**	**Samples**	**Abnormal**^**a**^	**Inherited/Polymorphism**^**b**^	**Phenotype**
Rooms (2004)	75	4 (5.3%)	-	Mental retardation
Koolen (2004)	210	8 (3.8%)	6 (2.9%)	Mental retardation
Northrop (2005)	51	3 (5.9%)	-	Mental retardation, congenital abnormalities
Kirchhoff (2005)	258	13 (5.0%)	16 (6.2%)	Mental retardation, dysmorphic features
Rooms (2006)	275	5 (1.8%)	7 (2.5%)	Mental retardation

^a ^Reported as likely to be clinically significant.

^b ^P019, P020 and/or P036A assays used, which contain some probes now known to detect polymorphic loci.

Although these studies demonstrated that MLPA was able to detect submicroscopic imbalance, few known abnormal loci have been tested by MLPA. Thus the sensitivity of MLPA and incidence of false negative results is unknown. This is of particular importance if diagnostic laboratories wish to replace multiprobe FISH with MLPA. The false negative rate will depend on the robustness of the MLPA technique itself and the analysis method used, either of which could result in miscalled negative results, and the experimental approach, which depending on the number of probes analysed may fail to identify a region of imbalance. In this paper, we describe the validation of MLPA for diagnostic testing in a service laboratory, including the testing of a large number of known abnormal loci, and the analysis protocol developed based on the validation data. We also review the results of the first 15 months of our diagnostic MLPA subtelomere testing service.

## Methods

### DNA extraction

DNA was extracted from blood samples received in EDTA using the Chemagic Automated DNA Separation System (Chemagen, Germany) following the manufacturer's instructions. DNA was also extracted in a minority of cases using the Puregene DNA extraction kit (Gentra Systems, USA) following the manufacturer's instructions or a salt-chloroform extraction method [17]. All DNA was quantified by fluorometry (Picogreen, Invitrogen, UK) or spectrophotometry (Nanodrop, USA) and checked for degradation on an agarose gel. Degraded DNA was not used for MLPA analysis.

### MLPA

Four MLPA subtelomeric probesets (MRC-Holland, The Netherlands) were used: P019 and P020 (which together test all the subtelomeres), P036 versions A & B (which each test all the subtelomeres) and P069 (which tests all the subtelomeres except those of the acrocentric chromosome short arms); P069 was released by MRC-Holland as a replacement for P019 and P020 during the course of the validation study. All patient samples were tested with a combination of P019+P020, P036A/B and P069, so that at least two loci were tested at each subtelomeric region. All probes for any subtelomeric region were analysed independently of each other. Any MLPA results for the 'short arms' of the acrocentric chromosomes were not considered significant as these probes hybridise to the long arm, near the centromere.

The MLPA kits were used to test 250 ng of DNA according to the manufacturer's protocol, with the following exceptions: (i) the PCR reaction was in a final volume of 25 μl; (ii) 26 PCR cycles were performed. Five normal male control samples were included for each MLPA assay, along with up to 88 patient DNA samples and one negative control.

A 3 μl aliquot of PCR product was mixed with 0.3 μl Genescan 500 LIZ size standard (Applied Biosystems, UK) and 15 μl HiDi formamide (Applied Biosystems, UK) before being size-separated by capillary electrophoresis on a 3100 genetic analyser (Applied Biosystems, UK). Genescan and Genotyper software (Applied Biosystems, UK) were used for fragment analysis, sizing and labelling of product peaks.

### MLPA data analysis

The peak height data was imported into a spreadsheet-based automated analysis system, which has been extensively validated. The system contained a series of quality checks to ensure that analysis was only performed when suitable data had been collected. These checks included ensuring that all peaks were present and labelled correctly, and that all peak heights were between 50–6000 arbitrary fluorescent units.

Dosage quotients were chosen to form the basis of the analysis where:

Dosage Quotient=height of patient test peakheight of patient reference peakmean(height of control test peakheight of control reference peak)
 MathType@MTEF@5@5@+=feaafiart1ev1aaatCvAUfKttLearuWrP9MDH5MBPbIqV92AaeXatLxBI9gBaebbnrfifHhDYfgasaacH8akY=wiFfYdH8Gipec8Eeeu0xXdbba9frFj0=OqFfea0dXdd9vqai=hGuQ8kuc9pgc9s8qqaq=dirpe0xb9q8qiLsFr0=vr0=vr0dc8meaabaqaciaacaGaaeqabaqabeGadaaakeaacqWGebarcqWGVbWBcqWGZbWCcqWGHbqycqWGNbWzcqWGLbqzcqqGGaaicqWGrbqucqWG1bqDcqWGVbWBcqWG0baDcqWGPbqAcqWGLbqzcqWGUbGBcqWG0baDcqGH9aqpdaWcaaqaamaaliaabaGaeeiAaGMaeeyzauMaeeyAaKMaee4zaCMaeeiAaGMaeeiDaqNaeeiiaaIaee4Ba8MaeeOzayMaeeiiaaIaeeiCaaNaeeyyaeMaeeiDaqNaeeyAaKMaeeyzauMaeeOBa4MaeeiDaqNaeeiiaaIaeeiDaqNaeeyzauMaee4CamNaeeiDaqNaeeiiaaIaeeiCaaNaeeyzauMaeeyyaeMaee4AaSgabaGaeeiAaGMaeeyzauMaeeyAaKMaee4zaCMaeeiAaGMaeeiDaqNaeeiiaaIaee4Ba8MaeeOzayMaeeiiaaIaeeiCaaNaeeyyaeMaeeiDaqNaeeyAaKMaeeyzauMaeeOBa4MaeeiDaqNaeeiiaaIaeeOCaiNaeeyzauMaeeOzayMaeeyzauMaeeOCaiNaeeyzauMaeeOBa4Maee4yamMaeeyzauMaeeiiaaIaeeiCaaNaeeyzauMaeeyyaeMaee4AaSgaaaqaaiabb2gaTjabbwgaLjabbggaHjabb6gaUnaabmaabaWaaSGaaeaacqqGObaAcqqGLbqzcqqGPbqAcqqGNbWzcqqGObaAcqqG0baDcqqGGaaicqqGVbWBcqqGMbGzcqqGGaaicqqGJbWycqqGVbWBcqqGUbGBcqqG0baDcqqGYbGCcqqGVbWBcqqGSbaBcqqGGaaicqqG0baDcqqGLbqzcqqGZbWCcqqG0baDcqqGGaaicqqGWbaCcqqGLbqzcqqGHbqycqqGRbWAaeaacqqGObaAcqqGLbqzcqqGPbqAcqqGNbWzcqqGObaAcqqG0baDcqqGGaaicqqGVbWBcqqGMbGzcqqGGaaicqqGJbWycqqGVbWBcqqGUbGBcqqG0baDcqqGYbGCcqqGVbWBcqqGSbaBcqqGGaaicqqGYbGCcqqGLbqzcqqGMbGzcqqGLbqzcqqGYbGCcqqGLbqzcqqGUbGBcqqGJbWycqqGLbqzcqqGGaaicqqGWbaCcqqGLbqzcqqGHbqycqqGRbWAaaaacaGLOaGaayzkaaaaaaaa@DFB0@

As dosage quotients are a relative measure, normalisation (transforming data points to have the same mean) was not necessary to take into account the differences in peak heights between samples.

Although peak heights have been used to calculate dosage quotients in this study, they can be successfully substituted with peak area measurements, as demonstrated by an evaluation of  >100 MLPA tests (data not shown). However, using peak heights has practical advantages as occasionally all peaks in a sample become very broad and Genescan analysis software may quantify peak area inaccurately. Peak height is not affected by this phenomenon and therefore, to maximise efficiency, it is preferable at our Centre to use peak heights for calculating dosage quotients.

A series of dosage quotients was generated for each peak by using individual neighbouring peaks as reference peaks, rather than generating a single dosage quotient for each peak by using an average of reference peaks. This avoids the situation whereby an abnormal peak may significantly alter the result of neighbouring peaks.

The control "test : reference" ratios were calculated for each of five controls and the mean of these ratios formed the denominator for the dosage quotient formula. Averaging the control ratios was necessary at this point to allow for inter-sample variability (including the differing degrees of tail-off observed between traces, discussed below). Coefficients of variation for the control "test : reference" ratios were calculated and each individual MLPA probe was considered valid only if there was less than 10% variation between the five control samples.

The normal range was defined as the square root of the expected theoretical ratios for deletions and duplications, i.e. √(0.5) – √(1.5), to avoid using subjective values. Importantly, this produces a normal range that is asymmetric around a 1:1 ratio, which reflects the biology. Each dosage quotient for a peak was called "deleted", "normal" or "duplicated" depending on where it fell in relation to the normal range. In addition, dosage quotients that were <0.5 or >1.5 were highlighted, as artefactual peaks occasionally occurred and were typically much larger or smaller than expected and hence gave very high or low dosage quotients.

**Figure 1:**
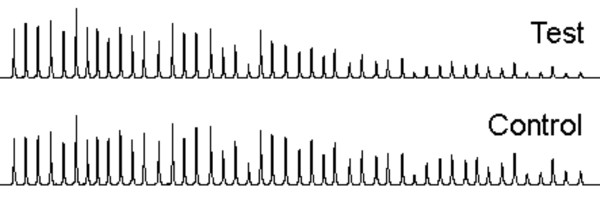
MLPA (P036B) traces for a normal test sample (upper) and normal control (lower) showing differential tail-off.

**Figure 2:**
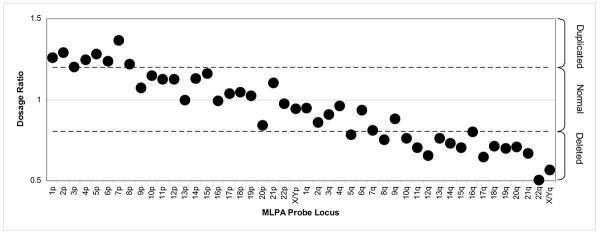
Analysis of the normal sample shown in Figure 1 using a global normalisation method (sample compared to 5 controls; normal range 0.8 to 1.2). A number of 'abnormal' results are generated.

**Figure 3:**
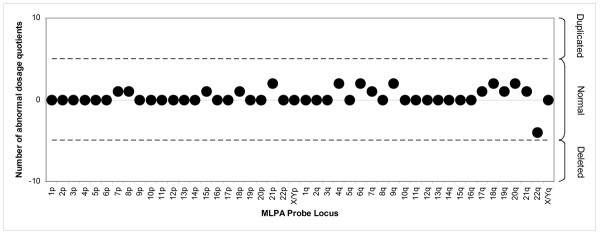
Analysis of the normal sample shown in Figure 1 using the method described in this paper (sample compared to 5 controls; normal range -5 to +5). All results are normal.

The modal (majority) call over the series of dosage quotients for each test peak gave the final result. An example of the dosage quotients generated for each sample is shown in table 2. To minimise the effects of differential tail-off mentioned above, a series of ten dosage quotients was generated for each probe by only using the ten neighbouring peaks as individual reference peaks. Reducing the amount of template DNA was not found to reduce the tail-off or differences in tail-off. Figure 1 illustrates a pair of traces, showing differences in tail-off between patient and control samples. Figure 2 illustrates the result of "global" normalisation (where each peak is normalised against all other peaks in that sample), whereas Figure 3 shows the same patient sample analysed by the method described in this paper. If more than 5 of the dosage quotients indicated a duplication or deletion, the result for the MLPA probe was classed as abnormal; abnormal results are however generally represented by 9 or 10 abnormal dosage quotients. Finally, the analysis system produced a summary table listing each MLPA probe for each patient and whether it indicated a deletion, duplication or normal copy number. For any sample exhibiting abnormal probe results, the test was repeated with the same assay to show consistency and any inconsistent results were rejected. Our spreadsheets are available on request.

**Table 2:** A section of the dosage quotient table generated for an abnormal sample.

6p	7p	8p	9p	10p	**11p**	12p	13p	14p	15p	16p
0.99	0.99	0.97	0.95	1.06	**0.49**	1.03	1.03	1.05	0.99	**2.00**
1.03	0.99	0.94	1.03	0.97	**0.51**	1.05	1.02	1.02	**2.00**	0.98
1.03	0.97	1.02	0.95	1.01	**0.52**	1.05	1.00	**2.04**	0.99	1.01
1.00	1.04	0.94	0.99	1.03	**0.52**	1.02	**1.99**	1.00	1.01	0.98
1.08	0.96	0.98	1.00	1.03	**0.50**	**2.03**	0.98	1.03	0.99	1.00
1.04	1.02	0.99	0.97	**1.99**	**0.49**	1.02	0.97	1.01	1.00	1.04
1.06	1.01	0.97	**1.94**	0.98	**0.50**	1.00	0.99	1.02	1.02	0.99
1.05	0.99	**1.92**	0.95	1.00	**0.49**	1.01	0.99	1.06	0.98	0.99
1.02	**1.97**	0.95	0.98	0.97	**0.50**	1.02	1.03	1.01	0.99	1.00
**2.04**	0.97	0.97	0.95	1.00	**0.50**	1.05	0.98	1.01	1.00	0.97

6p	7p	8p	9p	10p	**11p**	12p	13p	14p	15p	16p

N	N	N	N	N	**DEL**	N	N	N	N	N

The top and penultimate rows show the MLPA probe; each probe's 10 corresponding dosage quotients (generated from 10 flanking peaks) are shown in the column below. Abnormal ratios are highlighted in bold. The bottom row shows the result for each probe, where N signifies copy number of 2 and DEL signifies copy number of 1.

### Fluorescence in situ hybridisation

All subtelomeric abnormalities identified by MLPA were subsequently tested using single, chromosome-specific subtelomeric probes from Abbott-Vysis (ToTelVysion; UK), Cytocell (Aquarius; UK) or MP Biomedicals (QBiogene; CA, USA). Multisubtelomere FISH was carried out as previously described [18].

### Sequencing

DNA was sequenced using the BigDye Terminator sequencing kit (Applied Biosystems, UK) following the manufacturer's instructions. The sequencing products were analysed on a 3100 genetic analyser (Applied Biosystems, UK).

## Results

### Validation Study

124 patients, previously diagnosed with chromosome abnormalities were used in the validation study. A further 84 patients, referred for subtelomere screening during the course of this study, were also included. Therefore in total, 208 patients were tested, corresponding to 8528 genomic regions overall. Overall, 128 patients carried known abnormalities, corresponding to 184 abnormal regions and 415 tested abnormal loci. Validation cases included those with trisomy 13, 18 and 21, microscopically visible terminal deletions and duplications, sex chromosome abnormalities and submicroscopic abnormalities identified by multiprobe FISH. MLPA probes for all subtelomeric regions were validated for detecting normal copy number. Abnormal copy number detection was validated at the following subtelomeric loci: del 1p, 2q, 3p, 4p, 5p, 7p, 7q, 8p, 10p, 10q, 11p, 11q, 13q, 14q, 17q, 18q, 22q, Xp, Xq and dup 2q, 4p, 4q, 6p, 6q, 8p, 8q, 11p, 11q, 12p, 13p, 13q, 16q, 18p, 18q, 19q, 21p, 21q, 22p, Xp, Xq. All data collected during the validation study were analysed using the analysis method described above.

All known abnormal subtelomere regions were correctly identified by MLPA: false negative rate = 0; negative predictive accuracy = 1 (95% CI = 0.9996–1). In three cases with known abnormalities, the abnormality was only detected by one of the two MLPA probes, with the other probe hybridising to adjacent sequence of normal copy number: deletion of 10p subtelomere detected by P036A but not P019; deletion of Xp subtelomere detected by P036A but not P019; duplication of 16q subtelomere detected by P020 but not P036A. In all three cases, the more proximal probe detected normal copy number, further defining the breakpoint region.

One patient that had previously been diagnosed with monosomy 2q37 by G-banded chromosome analysis showed normal copy number for this region by MLPA. Further studies using FISH confirmed the MLPA result, and showed that the patient carried a balanced translocation between the long arm of chromosome 2 and the short arm of chromosome 5.

For one patient that was referred for subtelomere screening during the course of the validation study, a single MLPA probe (11p; P036A) indicated a deletion while the corresponding FISH test showed normal copy number. As other samples were available, seven members of this family were tested by MLPA. Interestingly, four affected family members across three generations exhibited the same deletion profile in the mucin 2 gene, whereas the three unaffected family members showed a normal profile. However, sequencing across the ligation site showed a sequence change, 2bp downstream from the ligation site (NM_002457.1:13593C>T) in all the "deleted" family members. The altered DNA sequence did not cause a change in the amino acid (isoleucine) and therefore the segregation of this sequence change with the phenotype is most likely to be coincidental in this family.

Another patient with a single MLPA probe deletion result (4q; P069) was also sequenced. This patient showed three base changes at the ligation site (NM_004477.2:133G>C, 134A>G, 135C>G). The MLPA ligation site for this probe corresponds to 5'-UTR of the FSHD region gene 1 (FRG1); further studies are required to determine whether these changes could alter the expression of the gene.

In addition, three other patients that had been referred for subtelomeric imbalance screening and that had been diagnosed as normal using FISH, were found by MLPA testing to carry abnormalities. In one of these cases, a single MLPA probe indicated a duplication (Xp; P019), while another more distal MLPA probe in the same region indicated normal copy number. The same probe indicated a duplication in the patient's mother who also had the same phenotype. We are waiting for samples from other family members to investigate this finding further. Parental samples have also been requested for the two other cases to help assess clinical significance; one interstitial duplication (12p; P020) which requires confirmation and one terminal duplication (Xp; P036A & P069).

In these five cases, the MLPA either gave or could be giving a false positive result in terms of dosage. Therefore the minimum positive predictive accuracy for MLPA dosage = 0.974 (95% CI = 0.951 – 0.996). However by using MLPA as part of an overall testing strategy that includes other techniques such as sequencing and FISH, false positive results can be avoided.

In summary, 184 known abnormal regions were identified by at least one of two MLPA probes. There were three novel abnormal findings, which require further follow-up testing to ascertain clinical significance. One patient was found to be balanced although previously diagnosed for monosomy 2qter. Therefore the validation study provided additional information for four patients, while no known abnormalities went undetected.

### MLPA diagnostic service

Between January 2005 and May 2006, 463 patients referred for subtelomeric imbalance screening were tested using MLPA. Due to the higher throughput of MLPA compared to multiprobe FISH, de Vries criteria [19] were not applied, but instead all clinical referrals from our region that requested subtelomere testing were screened. MLPA was carried out using the P036B and P069 kits; the probe loci in these two kits are different at each subtelomere.

Eight samples (1.7%) were not analysed as the DNA was degraded; these were all retrospective requests and the DNA had been extracted up to 10 years previously.

Out of the 455 patients tested, MLPA detected abnormal copy number in 27 patients (5.9%), summarised in Tables 3 and 4. One of these (case 8) consisted of both a duplication (22q; 1 probe) and a deletion (9q; 2 probes), which had not been detected by G-banded chromosome analysis. FISH analysis has confirmed the deletion and we are waiting for parental samples to aid assessing clinical significance. The abnormal results therefore comprised 16 single and 12 double MLPA probe results. Three of the single probe results were subsequently confirmed by another technique, either FISH (case 21) or by testing other family members (cases 20, 22). Sequence change at the ligation site was detected in a further patient (case 15, see below) and the remaining 12 unconfirmed single probe results are currently undergoing further studies.

**Table 3:** Summary of cases with two subtelomere MLPA probes indicating abnormal copy number.

**Case**	**MLPA result**	**FISH studies**^**a**^	**Inheritance studies**	**Clinical features**
1	del 1pter	-	De novo, parents unaffected	Developmental delay, mild dysmorphism

2	dup 1pter	dup 1pter	De novo, parents unaffected	Severe developmental delay, congenital heart defect

3	del 2qter	del 2qter	De novo, parents unaffected	Developmental delay, dysmorphism, IUGR

4	del 3pter	Normal, distal	-	Developmental delay, severe learning difficulties, spastic quadriplegia, epilepsy

5	dup 3pter	Normal, distal	Inherited from affected parent	Congenital heart defect, short stature, clinodactyly

6	dup 5qter	-	Inherited from unaffected parent	Queried Rubinstein-Taybi syndrome

7	del 6qter	-	Inherited from parent (unknown phenotype)	Severe developmental delay, microcephaly, no speech, seizures

8	del 9qter	del 9qter	-	Developmental delay, microcephaly, speech difficulties, seizures

9	dup 15qter	-	Inherited from parent (unknown phenotype), also present in another affected family member	Developmental delay, tall stature, macrocephaly, single kidney

10	dup 16qter	-	-	Developmental delay, cleft palate, hearing loss

11	del 17pter	-	-	Severe developmental delay, microcephaly, no speech, significant learning difficulties, mild dysmorphism, short stature, bilateral optic atrophy

12	dup 19qter	Normal, proximal	Sibling (phenotype unknown) carries same duplication, further inheritance studies to follow	Faltered growth, short stature, microcephaly

^a ^Result of FISH test and position of FISH probe relative to MLPA probes. No result indicates that no informative FISH probe or sample was available.

**Table 4:** Summary of cases with only one subtelomere MLPA probe indicating abnormal copy number.

**Case**	**MLPA result**^**a**^	**FISH studies**^**b**^	**Sequencing/qPCR**	**Inheritance studies**	**Clinical features**
13	del 1pter (P036B, proximal)	-	No sequence changes	-	Developmental delay, dysmorphism

14	del 1pter (P036B, proximal)	-	No sequence changes	-	Severe developmental delay, dysmorphism

15	del 4qter (P069, distal)	-	Three base changes next to ligation site	-	Severe developmental delay, microcephaly, finger contractures

16	del 4qter (P069, distal)	-	No sequence changes	-	Developmental delay, optic nerve hypoplasia

17	del 4qter (P069, distal)	-	No sequence changes^c^	-	Developmental delay, dysmorphism, arthrogryposis

18	del 4qter (P069, distal)	-	No sequence changes^c^	-	-

19	dup 6qter (P069, distal)	-	-	-	Cleft palate, micrognathia

20	dup 8qter (P036B, proximal)	-	-	Inherited from unaffected parent	Short stature, cardiac lesion, epicanthic folds

21	del 9pter (P069, distal)	del 9pter	-	To follow	Learning difficulties, microcephaly, heterochromatic irises, patches of hypopigmented hair, happy disposition

22	dup 9pter (P069, distal)	Normal, distal	-	Present in affected sibling, further studies to follow	Developmental delay, mild dysmorphism, speech delay, prominent teeth

23	dup 9qter (P069, distal)	Normal, distal	-	De novo, parents unaffected	Moderate learning difficulties, ventral-spetal defect, shaking, umbilical hernia

24	dup 13qter (P069, distal)	Normal, distal	-	-	Cleft palate, unilateral micropthalmia, coloboma

25	dup 15qter (P069, distal)	Normal, proximal	-	-	Congenital heart disease, cleft palate, short stature, ptosis

26	dup 22qter (P069, proximal)	-	-	De novo, parents unaffected	Developmental delay, autoimmune liver disease

8	dup 22qter (P036B, distal)	Normal, proximal	-	-	Developmental delay, microcephaly, speech difficulties, seizures
27	dup 22qter (P036B, distal)	Normal, proximal	-	De novo, parents unaffected	Developmental delay, hypospadias

^a ^MLPA result, probeset and position of abnormal MLPA probe relative to normal MLPA probe.

^b ^Result of FISH test and position of FISH probe relative to abnormal MLPA probe. No result indicates that no informative FISH probe or sample was available.

^c ^Homozygous/hemizygous single base change detected 24 bp from ligation site.

There were 13 cases with deletion profiles, three of which were shown to be interstitial (cases 4, 13, 14); duplications made up 15 cases, six of which were shown to be interstitial (cases 5, 20, 22, 23, 24, 26).

Further family members were tested for 13 of the 28 abnormal results: six of these (cases 1, 2, 3, 23, 26, 27) were found to be *de novo *abnormalities, although cases 23, 26 and 27 were single probe results and need to be confirmed; seven have been shown to be inherited. Of these seven, the abnormality detected by MLPA had been inherited from unaffected parents in two patients (cases 6 and 20). In a further three cases (5, 9 and 22), the same abnormality was detected in affected family members. In the remaining two cases (7 and 12), it remains unclear whether the abnormality was segregating with phenotype.

Seven of the 16 single MLPA probe results indicated deletions, four of which were detected by the 4q probe of the P069 assay. One (case 21; 9p, P069) was confirmed by FISH analysis. The other six cases were sequenced. Five showed normal sequence (cases 13, 14, 16, 17 and 18). One (case 15; 4q, P069) showed three heterozygous sequence changes in the FRG1 gene (NM_004477.2:133G>C, 134A>G, 135C>G). Interestingly this was the same sequence change as that detected in one of the patients in the validation study (see above).

## Discussion

Previous publications [11,12,14] on the use of MLPA to detect subtelomere imbalance recommended the use of two different probes to identify abnormal subtelomeric regions; both MLPA probes needed to demonstrate concordance for an abnormal result to be called. However, imbalance identified by only a single probe may be a true positive result, as although some MLPA probes are only 4 bp apart (4q subtelomere), they can be as much as 1.2 Mb apart (13q subtelomere) and have a median separation of ~40 Kb. Furthermore, regions of imbalance may stretch away from the second locus tested when the breakpoint lies between the two MLPA probes. Use of only concordant two-probe results will therefore inevitably result in some false negative results, as illustrated by three cases in our validation study and three cases from our diagnostic service. We therefore developed an analysis strategy that generates reliable single probe results, an important development for the introduction of MLPA into our clinical diagnostic service.

Our analysis model was developed to minimise the effects of differential tail-off, to remove bias caused by neighbouring abnormal loci, and to have defined parameters for calling deletions and duplications. A number of analysis approaches were investigated with these aims in mind, in addition to avoiding false negative single probe results due to technical or analytical artefacts, and minimising analogous false positive results. Analysing MLPA data involves comparing a test peak to a reference peak to detect abnormal copy number. One method, initially proposed by MRC Holland and used by Northrop *et al *[11] and Koolen *et al *[10], normalised each peak against an average of all peaks for that sample (global normalisation), and then generated a dosage ratio by comparison to normalised control data. However, this method does not account for the observed peak tail-off, where larger MLPA products exhibit reduced peak heights compared to smaller products, and in particular the variation in tail-off between samples (both test samples and controls; see Figure 1) that appears to be unpredictable.

In the course of this study, extensive peak tail-off was observed in a minority of samples, and in these cases, global normalisation caused dosage ratios to vary away from 1 (Figure 2). When global normalisation was used in a previous study [10], ~18% of MLPA traces were failed. To minimise the effect of this differential tail-off, others, including Kirchoff et al [12], normalised the test peak against the average of a small number of neighbouring peaks (local normalisation). However, any abnormal peak will skew the results of the peaks that use it for normalisation. If this effect is substantial, then this can cause a false-positive result.

**Figure 4:**
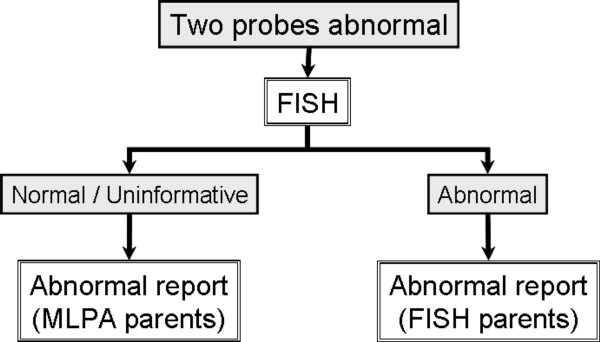
MLPA workflow for abnormal results in cases with two probe results.

**Figure 5:**
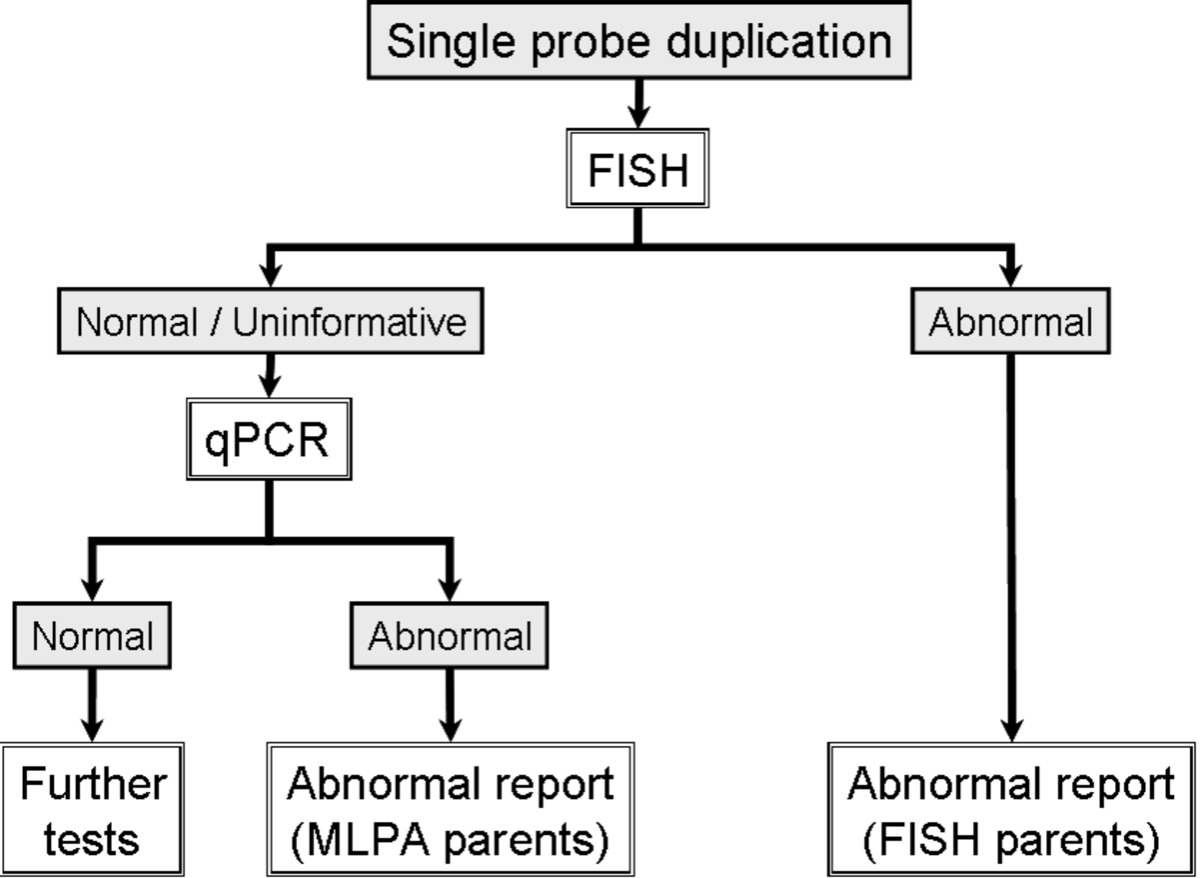
MLPA workflow for abnormal results in cases with single probe duplications.

**Figure 6:**
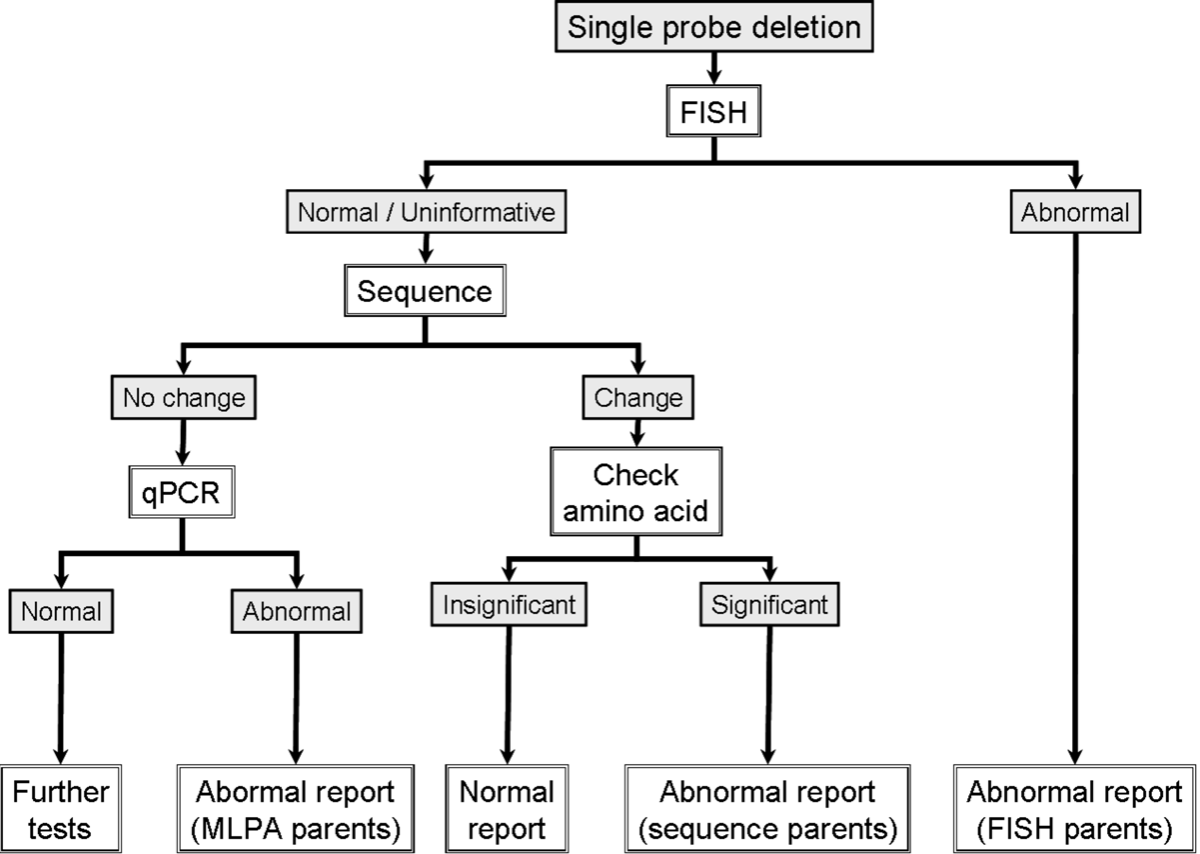
MLPA workflow for abnormal results in cases with single probe deletions.

The analysis method described in this paper, developed and validated on a dataset of ~20,000 tested loci (208 cases, each tested two or three times), minimises any manipulation of data (values are not pooled or averaged) and considers each result independently of other results from the same subtelomeric region. This method generates robust single probe results as shown by the data set presented here. However, best practice still requires that any result based on a single locus is confirmed by a separate assay, either for the same locus, or for a nearby region. We have chosen to follow up single probe results using FISH, sequencing and/or realtime PCR, according to the flow diagrams shown in Figures 4, 5 and 6. It is interesting to note that in a separate case with a deletion profile, a single base change 10 bp from the ligation site was identified (data not shown). As the identification of any heterozygous sequence change in the probe binding site provides evidence for the presence of two homologues, this suggests that a single base change up to 10 bp away from the ligation site can affect probe ligation and/or hybridisation efficiency sufficiently to simulate a deletion.

Inheritance studies are required to determine if an abnormality is *de novo*, which would suggest that it is associated with the abnormal phenotype. However, an inherited abnormality is not necessarily unconnected to the referral indication, and careful clinical assessment of the carrier parent's phenotype is necessary to exclude subtle abnormalities or unusual features. In addition, recent studies [20-22] have shown that the incidence of "polymorphic" copy number variations (CNVs) in the genome is higher than had previously been thought. Although some of the MLPA probes in the subtelomeric assays have been redesigned to account for CNVs, it is likely that MLPA assays may continue to detect rare CNVs such as cases 6 and 20 reported here. However, the possibility that CNVs may be important in complex disease or susceptibility traits cannot be excluded, and the collection and collation of these findings will be an important contribution to our understanding of human variation. In addition, two samples (one from the validation study and service case 15) both carry the same 3 bp change near the ligation site of a MLPA probe for the 4q subtelomere (P069). This is therefore likely to represent a population polymorphism although further studies are required to exclude expression of FRG1 being affected by this change in the 5'-UTR sequence.

Overall, the 27 abnormal MLPA results found in the 455 patients tested as part of our clinical diagnostic service (see Tables 3 and 4), represent an abnormality detection rate of 5.9%, although some of these have yet to be confirmed, and some were inherited from apparently normal parents, and may therefore be incidental findings. The prevalence of "clinically significant" abnormalities may therefore be as low as 1.3% and as high as 5.5%. Prevalence of subtelomere imbalance has previously been reported as between 0 [1] and 10.7% [2], by FISH and previous MLPA studies. Detection rates in different studies are likely to be influenced by two factors: first, the patient group tested, and second, the resolution and quality of the G-banded chromosome analysis carried out prior to MLPA testing. Our tested group is now less phenotypically defined than prior to MLPA, as we are able to test many more samples, and no longer restricted to patients fulfilling the de Vries criteria [19]. We would therefore expect our detection rate to be lower than other groups still using these criteria. In addition, our G-banded chromosome analysis is routinely carried out at a minimum quality of 550 bands per haploid set, and usually at a higher quality. This enables us to detect subtle abnormalities in the subtelomere regions and exclude these from MLPA testing. A policy of "high resolution" chromosome analysis with particular emphasis on the examination of telomeres has previously been shown to reduce the detection rate by FISH of subtelomere abnormalities [1].

### Testing Strategy

The results of the validation study were used to develop a testing strategy, which is now in use for our MLPA service (see Figures 4, 5, 6). All samples are tested with 2 different MLPA probes for each subtelomere and any abnormal results are repeated to check for consistency. Naturally, if the breakpoint for the abnormality occurs between the two MLPA probes, only one probe will produce an abnormal result. All abnormal MLPA results are routinely followed-up with FISH analysis, which may confirm the MLPA result, and/or provide positional information and delineate breakpoints. However, as the relative positions of commercial FISH and MLPA probes vary between regions, FISH analysis may not be informative in some cases. Developing further FISH probes could be one strategy for aiding follow-up studies.

For samples where only one MLPA probe gives an abnormal result, confirmation by another technique is considered necessary before reaching a conclusive diagnosis. As mentioned above, FISH analysis is the first follow-up test. When deletions identified by a single MLPA probe are not confirmed by FISH (or FISH would be uninformative due to relative probe positions), these cases are then followed up by sequencing across the MLPA probe binding site (see Figure 6). This identifies any sequence changes near the ligation site, which can inhibit ligation of the MLPA probe and simulate a deletion. If any heterozygous sequence change is detected in the probe binding sequence, then, regardless of the position of the sequence change, this indicates the presence of two copies of this sequence. The deletion detected by MLPA is therefore either due to this sequence change or an artefact of the MLPA reaction. In either case, it is unlikely to be clinically significant, although a sequence change which may give rise to an amino acid change in a critical region of a protein should be considered. If no sequence change is detected, another technique is still required to confirm the deletion. Quantitative realtime PCR is a promising approach for this role and is currently in development at our Centre. Similarly, duplications detected by a single MLPA probe require confirmation (see Figure 5) and again, realtime PCR assays are being developed for this purpose for instances when the FISH result is normal or would be uninformative.

Parental samples are requested for any patients with confirmed abnormal results to assess the clinical significance of the findings. In addition, MLPA analysis of parental samples may confirm single probe MLPA results if the deletion (normal on sequence analysis) or duplication is inherited.

## Conclusion

MLPA is an extremely efficient, medium-thoughput technique; we routinely test 80 samples per week (including patients from other referral groups not detailed here), and on the grounds of both throughput and cost MLPA has very considerable advantages over FISH. The detection of imbalance across the genome by new methods such as array-CGH will clearly increase the detection rate of abnormalities in patients with developmental delay, dysmorphism and mental retardation. However, array-CGH is unlikely to be introduced into diagnostic service in a state-funded health system for this substantial referral group until the cost of the arrays is considerably less than at present and/or cytogenetic services have been rationalised; until then, MLPA represents the best available approach for detecting submicroscopic imbalance in regions known to be gene-rich and prone to rearrangements. The development of a straightforward and robust analysis system for the interpretation of results from the subtelomere assays will be of benefit to all Centres wishing to implement this service.

## Competing interests

The author(s) declare that they have no competing interests.

## Authors' contributions

JWA participated in the design of the study, designed the statistical analysis and drafted the manuscript. CO participated in the design of the study and helped to draft the manuscript. AW and HT carried out the molecular genetic studies. RM participated in the sequence alignment. AH and CD performed the statistical analysis. KM participated in the design of the study and helped to draft the manuscript. All authors read and approved the final manuscript.

## Pre-publication history

The pre-publication history for this paper can be accessed here:


